# Antigen-Specific TCR-T Cells for Acute Myeloid Leukemia: State of the Art and Challenges

**DOI:** 10.3389/fonc.2022.787108

**Published:** 2022-03-09

**Authors:** Synat Kang, Yisheng Li, Jingqiao Qiao, Xiangyu Meng, Ziqian He, Xuefeng Gao, Li Yu

**Affiliations:** ^1^ Department of Hematology and Oncology, International Cancer Center, Shenzhen Key Laboratory of Precision Medicine for Hematological Malignancies, Shenzhen University General Hospital, Shenzhen University Clinical Medical Academy, Shenzhen University Health Science Center, Shenzhen, China; ^2^ Central Laboratory, Shenzhen University General Hospital, Shenzhen, China

**Keywords:** acute myeloid leukemia, TCR-T cells, immunotherapy, allo-HSCT, immune escape

## Abstract

The cytogenetic abnormalities and molecular mutations involved in acute myeloid leukemia (AML) lead to unique treatment challenges. Although adoptive T-cell therapies (ACT) such as chimeric antigen receptor (CAR) T-cell therapy have shown promising results in the treatment of leukemias, especially B-cell malignancies, the optimal target surface antigen has yet to be discovered for AML. Alternatively, T-cell receptor (TCR)-redirected T cells can target intracellular antigens presented by HLA molecules, allowing the exploration of a broader territory of new therapeutic targets. Immunotherapy using adoptive transfer of WT1 antigen-specific TCR-T cells, for example, has had positive clinical successes in patients with AML. Nevertheless, AML can escape from immune system elimination by producing immunosuppressive factors or releasing several cytokines. This review presents recent advances of antigen-specific TCR-T cells in treating AML and discusses their challenges and future directions in clinical applications.

## Introduction

Acute myeloid leukemia (AML), which is a relatively common leukemia in adult patients, results from aberrant growth in the hematopoietic system, and it has multiple clinical appearances (1, 2). Complete remission for AML remains difficult to achieve despite recent advances in chemotherapy and molecularly targeted therapies (3), and chemotherapy is the first-line treatment option for AML patients. The 5-year survival rates of patients below the age of 60 years are 30% to 35% and less than 15% for those aged 60 years and above (4, 5).

Allogeneic hematopoietic stem cell transplantation (allo-HSCT) remains the only established curative strategy for some types of relapsed or refractory AML (6–8). Analyses of adult AML patients have revealed that allo-HSCT treatment prior to the first complete remission resulted in a reduction of the risk of disease relapse by more than 60% compared with chemotherapy alone (9). Similarly, several studies using haploidentical donors have shown therapeutic effects on the first complete remission of 34% to 47% (10, 11). Moreover, patients receiving allo-HSCT demonstrated significantly higher OS than patients receiving chemotherapeutic postremission therapy (12). However, the results from these modest adoptive cell therapy (ACT) strategies for AML remain unsatisfactory due to high rates of graft-vs.-host disease (GVHD) and relapse (6), which could be explained by immune escape reasons (13–16).

**Figure 1:**
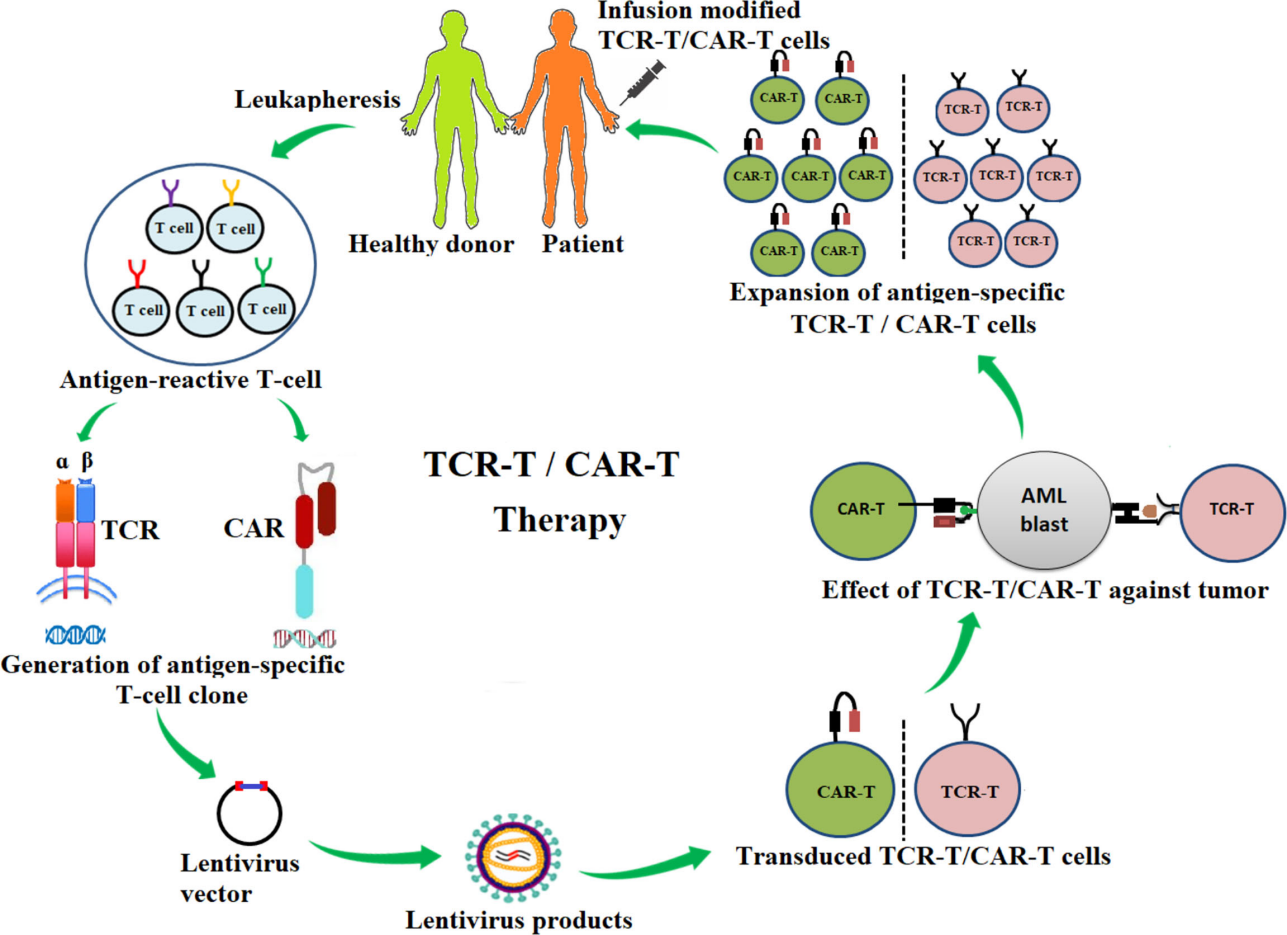
Schematic diagram of the adoptive transfer of antigen-specific T-cell receptor redirecting T cells (TCR-T) and chimeric-antigen receptor redirecting T cells (CAR-T) for AML immunotherapy. Antigen-specific T-cell clones are generated from antigen-reactive T cells of healthy donors or patients and are inserted into a lentivirus vector. The lentivirus vector is transfected into the packing cells for the production of lentiviral particles. The lentiviral particle products containing desired αβ-TCR or CAR genes are then used to infect T cells (TCR-T, CAR-T). These genetically modified TCR-T or CAR-T cells are tested for effectiveness against cancers. TCR-T or CAR-T products are then expanded *in vitro* and infused into patients.

ACT with antigen-specific T cells, including chimeric antigen receptor T cells (CAR-T cells) and T-cell receptor-engineered T cells (TCR-T cells), involves the generation and modification of targeted T cells. These therapies have shown high potency against diverse tumors, including AML **(** **Figure 1** **)** (14, 17–30). The FDA approved the first CD19 CAR-T-cell product, KYMRIAH (tisagenlecleucel), to treat acute lymphoblastic leukemia in children and adults (19). CD19 CAR-T cells are widely used for treating hematological cancers, especially leukemia (20–22). Two clinical trials exploring the use of CD19 CAR-T in the treatment of AML are currently recruiting (NCT04257175, NCT03896854). Clinical trial NCT04257175 is a phase II/III, while clinical trial NCT03896854 is a phase I/II trial in which the primary goal is to measure adverse events. Importantly, the use of second-generation autologous CD123 CAR-T cells has demonstrated a potent efficacy (NCT02159495). Here, six patients were enrolled in the study and were administered various doses of CD123 CAR-T cells: two patients received 5.0 × 10^7^ CD123 CAR-T and four patients received 2.0 × 10^8^ CD123 CAR-T cells. One of the patients who was treated with the lower dose of cells experienced a reduction of leukemia blast counts (from 77.9% to 0.9%), while one of the patients who was treated the higher dose achieved complete remission. The other three patients experienced reductions of blast counts but not complete remission (23).

In addition to the use of CAR-T cells, the success of adoptive transfer of antigen-specific TCR-T cells in murine studies was originally reported by Dembic et al. in the late 1986. Here, α and β TCR genes were transduced into T cells in order to enhance recognition of antigen-specific peptides presented by major histocompatibility complex (MHC) I (31). Subsequently, αβ-TCR-T cell specific for melanoma antigen recognized by T cell (MART)-1 were generated by Clay et al., who found that the redirecting of human peripheral blood lymphocytes (PBL) with αβ TCR efficiently allowed for recognition of a peptide antigen specific to melanoma cells (32). In addition, adoptive transfer of autologous TCR-T cells specific for New York esophageal squamous cell carcinoma (NY-ESO**)**-1 has resulted in remarkable clinical responses and a safe profile in the treatment of several cancers, including melanoma, synovial cell sarcoma, and nonsmall cell lung cancer (24–27). Treatment with autologous T cells transduced with NY-ESO-1 TCR with an increased affinity has achieved clinical responses in 80% of patients with myeloma (33).

Several relevant breakthroughs in leukemia immunotherapy have been reported over the past few years (28–30). Treatment of high-risk AML patients with adoptive transfer of Wilms’ tumor antigen 1 (WT1)-specific allogeneic TCR-T cells has shown promising results and helped prevent relapse (28). Another clinical trial (NCT02550535) of autologous WT1-specific TCR-T cells was performed to assess treatment of high-risk myeloid malignancies, and it demonstrated strong efficacy with a good safety profile (30). These findings highlight the potential for TCR-T cell therapies to improve outcomes in AML. Unfortunately, cancer cells produce several immunosuppressive factors that facilitate escape from detection by the immune system.

In this review, we present the state of the art and challenges of antigen-specific TCR-T cell immunotherapy for managing myeloid malignancies and discuss future directions of TCR-T for treating AML.

## The Current State of TCR-T Cell Immunotherapy for AML

### TCR-T Cell Immunotherapy for AML in Preclinical Studies

Selecting the appropriate target is crucial for the success of TCR-T therapy. Optimally, the antigen target must be highly overexpressed in cancerous cells but have limited expression in the healthy hematopoietic system. However, if the target antigen is expressed in normal blood cells, it must be at a low level, it must be dispensable in normal cells, and it must not be displayed as a human leukocyte antigen (HLA). If the target antigen is expressed in normal blood cells, short-lived TCR-T must be used for targeting (34). Thus far, several types of tumor-associated antigens (TAAs) and other potential targets have been reported in preclinical studies of TCR-T therapy in AML **(** **Table 1** **)**. WT1, minor histocompatibility A (HA)-1, telomerase (TERT), and survivin, neoantigens, and cancer-testis antigens (CTAs) have been reported as TAAs and have been explored in preclinical trials (34, 50). TAAs have been classified into several categories: overexpressed antigens (e.g. survivin and TERT), lineage-restricted antigens (e.g., WT1), cancer-testis antigens (e.g., NY-ESO-1, MAGE, and PRAME), neoantigens (e.g., NPM1 and CBFB-MYH11), and HA-1 (34, 50, 51).

**Table 1:** Adoptive transfer of antigen-specific TCR-T cells against AML in the preclinical study.

Tumor-associated antigens	Antigen-specific TCRs	Types of T cells	Manipulation	HLA restriction	Effect of TCR-T against AML	References
Overexpressed antigens	TERT TCR-T	T cells	High-affinity TCR	HLA-A*0201	Efficiently lysed primary and AML cell lines *in vitro* and inhibited tumor growth prolong survival rate of AML xenograft model.	(35)
	Survivin-TCR-T	CD8^+^ T cells	Codon optimization of TCRs	HLA-A*0201	Specifically lysed AML *in vitro*.	(36)
Lineage-restricted antigens	WT1 TCR-T (NTLA5001)	CD4^+^ T cells, CD8^+^ T cells	CRISPR/Cas9 genome editing	HLA-A*02:01	High effectiveness in controlling tumor growth and increased survival in the animal model. No GVHD was observed.	(37)
	WT1 TCR-T	T cells	High-affinity TCRs	HLA-A*02:01	Highly lysed fresh BM or PBL of AML blasts and eliminated AML in xenograft	(38)
	WT1 TCB-T	T cells	TCR-like TCBs combining with lenalidomide	HLA-A*02:01	Mediated killing primary AML *in vitro* and animal model.	(39)
Minor histocompatibility antigens	HA1 TCR-T	CD4^+^ T cells, CD8^+^ T cells	iCasp9 genome editing	HLA-A*02:01	Potential killing cell lines and primary relapsed/refractory AML or LCL.	(40)
	HA1 TCR-T	T cells	Codon optimization of TCRs	HLA-A*02:01	Increased cytolytic function against AML/LCL.	(41)
	mHagHA-2-TCR, TCR-mHag DBY, CMV pp65-TCR	γð T cells	αβ TCR transduced γð T cells	HLA-A*0201	Highly lysed primary AML blasts.	(42)
				HLA-B*07:02		
				HLA-DQ5		
Cancer-testis antigen	PRAME TCR-T	T cells	High-avidity TCRs	HLA-A*02:01	High efficacy lysis several tumor cells, including primary AML blasts.	(43)
Neoantigens	NPM1 TCR-T	CD4^+^ T cells, CD8^+^ T cells	Codon optimization of TCRs	HLA A*02:01	Specifically killed AML cell lines and primary AML blasts and controlled tumor outgrowth and prolonged survival in a xenograft model.	(44)
	CBFB-MYH11 TCR-T	CD8^+^ T cells	High-avidity TCR	HLA-B*40:01	Potent antileukemic activity against primary AML cells and in xenograft model.	(45)
	HMMR-TCR	T cells	High-affinity TCR	HLA-A*0201	High-effective controlling solid tumor growth and hematopoietic malignant such as AML.	(46)
	MDM2-TCR	CD8^+^ T cells	High-affinity TCR	HLA-A*0201	Highly lysed the specific target cells.	(47)
	FMNL-TCR	CD4^+^ T cells	DC-pulsed FMNL1	HLA-DRB1*0101, HLADRB1*1101	Increased several cytokines release again AML *in vitro.*	(48)
	HLA-DPB1 TCR	CD4^+^ T cells	Codon optimization of TCR	HLA-DPA1*01:03, HLA-DPB1*04:01	Highly lysed AML *in vitro* and xenograft model.	(49)

#### Overexpressed Antigens

##### Telomerase TCR-T

Telomerase (TERT) is a ribonucleoprotein enzyme that acts as an organizer at the ends of eukaryotic chromosomes. It is expressed and activated under the control of multiple regulatory mechanisms, which include trafficking and posttranscriptional and posttranslational modifications, to maintain homeostasis of telomere lengths. Alterations to these regulatory mechanisms result in the dysfunction of telomeres and the development of multiple human diseases (52).

TERT is absent in most human somatic tissues but is expressed highly in most AML patients (~85%) and is most highly expressed in patients with relapsed AML (35). An earlier study demonstrated cytolytic activity of TERT-specific cytotoxic T lymphocytes (CTLs) against several cancers, including leukemia (53–55). TERT-specific CTLs, which were generated by stimulating CTLs with artificial antigen-presenting cells (APCs), have also been demonstrated to have cytotoxic activities against solid tumors and hematopoietic malignancies (55). Increasing antitumor activities were associated with expression of TERT and HLA serotype A*02:01 by the target cells (55). Moreover, adoptive transfers of high-avidity TERT-specific TCR-T cells in the context of HLA-A*02:01-restricted targets have been shown potential for controlling tumor growth and prolonging the survival of tumor-bearing mice in AML (35). However, the targeting of AML by TERT-specific TCR-T cells has not yet been evaluated clinically. Intriguingly, several clinical trials of a TERT-peptide vaccine have been shown to have activity against several cancers, including nonsmall cell lung cancer, prostate cancer, and multiple myeloma (56–59).

##### Survivin TCR-T

Survivin, which is encoded by the *BIRC5* gene, plays an essential role in inhibiting apoptosis, regulating the cell cycle, and regulating the anti-tumor activities of T cells (60). Survivin is not expressed, or is expressed at very low levels, in normally differentiated cells (60), but it has been found to be highly expressed in various cancers, including AML (61–65). Survivin-specific CTLs in the context of HLA-A2 restriction have been demonstrated to efficiently lyse diverse types of tumor cell lines and primary leukemia cells, including those from AML, acute lymphoblastic leukemia (ALL), and chronic lymphocytic leukemia (CLL) (66). Stimulating T cells with dendritic cells expressing survivin-specific mRNA have been shown to be effective against an AML patient-derived blast and xenograft model (67). Moreover, survivin-specific CTLs have been demonstrated to sufficiently recognize and kill survivin- and HLA-A2-positive leukemia cells in patients with AML, without cross-reactivity against healthy progenitor cells (68). Subsequently, Arbor et al. (36) generated survivin-specific TCR-T cells in the context of HLA-A*02:01. These cells were shown to avoid fratricidal effects or toxicity during normal hematopoietic stem-cell transplantation (HSCT). Survivin-specific TCR-T cells also have been shown to have high specificity and efficacy against AML targets without on-target, off-tumor toxicity. Notably, in *in vivo* studies, survivin-specific TCR-T showed potent antitumor activity and prolonged survival in a xenograft mouse model (36).

#### Lineage-Restricted Antigens

##### WT1 TCR-T

The gene encoding WT1 is located on chromosome 11p13. The protein includes an N-terminal domain and a C-terminus containing four zinc-fingers that are organized as multiple isoforms (69, 70). Different isoforms tend to be differentially expressed in patients with relapsed AML (69). WT1 is an intracellular antigen highly expressed in the bone marrow of patients with leukemia, particularly those with AML, myelodysplasia (MDS), and CLL (39, 69, 71). WT1 is an ideal target for cancer immunotherapy due to its limited expression on healthy tissues. The success of WT1-specific CTLs and WT1-specific TCR-T cells for eliminating leukemia cells was demonstrated *in vitro* and in xenograft models several years ago (38, 72). WT1-specific CTLs showed specific cytotoxicity against leukemia cells and achieved sustained remission in patients with refractory AML (73). Moreover, studies of high-avidity WT1-specific TCR-transduced CTLs in the context of HLA-A*02:01 were conducted, and they demonstrated a high degree of lysis of CD34^+^ cells in fresh bone marrow or blood samples from AML patients and the potential elimination of leukemia blast cells in xenograft models (38). In addition, a WT1-specific TCR-like T-cell bispecific antibody (TCB) redirecting T cells showed enhanced efficiency in killing AML cell lines and primary AML cells (39).

A concern regarding therapies that involve the adoptive transfer of TCR-T cells is that mispairing of introduced and endogenous TCR chains may decrease the avidity of T cells against primary cancers and subsequently lead to the presentation of low levels of relevant peptides on cell surfaces (74, 75). Therapeutic strategies must, then, avoid such mispairings and competition with endogenous TCR α and TCR β chains, which could result in off-tumor toxicity and GVHD or negatively impact T-cell specificity and TCR expression levels. To that end, Ruggiero et al. (37) created high avidity of WT1 TCR-T cells modified through a strategy involving CRISPR/Cas9 to eliminate the endogenous TCR α and TCR β chains. Resulting WT1-specific TCR-T cells exhibited high efficacy in killing primary AML from bone marrow and ALL tumor-bearing NOD SCID gamma mice (37). The treatment of these mice with genetically modified WT1-specific TCR-T cells significantly reduced tumor growth and enhanced survival without inducing GVHD (37). Rather than using CRISPR/Cas9, Fujiwara et al. (76) alternatively generated appropriately modified T cells with both WT1-specific TCRs and siRNAs (siTCRs) to avoid the primary concern of autoimmune reactivity caused by mispairing between introduced and endogenous TCR chains with unknown specificity (76). WT1_235-243_-specific siTCR-T cells in the context of restricted HLA-A*24:02 were shown to have significantly enhanced antileukemia efficacies, and they extended animal survival. These positive results were associated with the presence of memory T cells in the mice modified with WT1-siTCR/CD8^+^ T cells (76). Thus, preclinical studies of WT1-specific TCR-T cells demonstrated advanced benefits; clinical studies of WT1-specific TCR-T cells will be discussed further, below.

#### Minor Histocompatibility Antigens

##### HA-1 TCR-T

HA-1 is a peptide of nine amino acids encoded by a diallelic gene on human chromosome 19 (77). Significant differences in the immunogenicity of the HA-1 T-cell epitope can be traced to the identity of the amino acid at position 3 (i.e., VL**H**DDLLEA genotype RS_1801284 A/G or A/A vs. VL**R**DDLLEA, genotype RS_1801284 G/G) (77). Between these two peptides, the HA-1^H^ (VL**H**DDLLEA) peptide can only be presented on the cell surface with highly HLA-A*0201-restricted CTLs (78), while the HA-1^R^ (VL**R**DDLLEA) peptide cannot be delivered to the cell surface, even though both nanopeptides can bind to HLA-A*0201.

The HA-1^H^ (hereafter referred to as HA-1) antigen is abundantly expressed in leukemia and normal hematopoietic cells, but its expression is restricted in nonhematopoietic cells (34). In HA-1-mismatched HCT, the HA-1^−^ donor immune system is not tolerant to HA-1 because it is considered self-antigens (40). A study of HA-1-specific CD8^+^ CTL showed that APCs coated with HLA-A*02:01/HA-1 stimulated CD8^+^ CTL (donor-derived HLA-A*02:01/HA-1^−^) to kill HA-1-positive cells in primary leukemia blasts (79).

Based on this success, additional approaches to generate HA-1-specific TCR redirecting T cells have been developed (41, 80). The transduction of PBL or cord blood (CB) with HA-1-specific αβ TCR demonstrated cytolytic activity against HLA-A2^+^/HA-1^+^ of AML and lymphoblastoid cell lines (LCLs). However, the detection of HA-1 TCR-positive cells showed a low level of HA-1-specific tetramer affinity due to the mismatched TCR structure between exogenous TCR and endogenous TCR (80). The affinity of HA-1-specific TCR has been improved by TCR codon optimization to increase TCR expression on the cell surface (41). HA-1-specific TCR-T showed efficient expression in transduced TCR and enhanced HA-1-specific functional activity against primary AML cells and LCL lines (41). Moreover, high-affinity HA-1-specific TCR-T cells containing an inducible caspase 9 safety switch, generated from the repertoire of a healthy HLA-A*02:01-positive HA-1-negative cell, have demonstrated high efficiency in killing HA-1^+^ primary AML and LCL (40). Notably, the coexpression of CD8 receptor and high-affinity HA-1-TCR by CD4^+^ cells led to specific killing of HA-1-containing target cells without cross-reactivity (40).

#### Cancer-Testis Antigens

##### CTA-Specific TCR-T

CTAs are a group of TAAs that exhibit normal expression in the adult testis but aberrant expression in several types of cancers (51). So far, more than 200 CTA genes from 44 gene families have been found to be encoded in the human genome *via* analysis of the CTdatabase (51). These CTAs can be classified into two groups depending on whether they are localized to the X-chromosome (Xq21-q28) or to non-X-chromosomes (51). Chromosome X-encoded CTAs include melanoma antigen (MAGE), NY-ESO-1, G antigen (GAGE), CT45, and synovial sarcoma, X chromosome (SSX), whereas non-X CTAs, located on autosomes, include B melanoma antigen (BAGE), helicase antigen (HAGE), and sperm protein 17 (SP17) (81, 82).

The CTA expression level mainly depends on the tumor type, the degree of differentiation, and the stage of progression. CTAs are potential targets for adoptive T-cell therapy because they are not expressed in normal somatic tissues accompanied by their relatively high expression in malignant cancers and their re-expression in several tumors (83). Immunotherapies targeting CTAs, including NY-ESO-1, MAGE-A3, and preferentially expressed antigen in melanoma (PRAME), have demonstrated high antitumor efficacies (84, 85). PRAME-specific CTLs in the context of HLA-A*02:01-restricted epitope have been generated from AML patients after allo-HSCT (86). Moreover, high-avidity PRAME-specific TCR-T cells generated from severe GVHD after HLA-mismatched HSCT have demonstrated high efficacy against a wide variety of tumor cell lines and AML primary cells (43). Multileukemia antigen-specific T cells, which included TCRs against PRAME and MAGE-A3, have shown antitumor reactivity against AML blasts (87). Accordingly, clinical testing of the utilization of *ex vivo*-stimulated HSCT donors against PRAME, MAGE-A3, and other tumor-associated antigens (WT1, NY-ESO-1, and survivin) is ongoing (NCT02494167 and NCT02203903).

#### Neoantigens

##### Neoantigen TCR-T

Neoantigens, which are highly immunogenic, are found in several solid tumors and hematopoietic malignancies, including AML (88, 89). Neoantigens can be divided into shared neoantigens and personalized (uniquely mutated) neoantigens (90). Shared neoantigens are mutated antigens that are common across different cancer patients but are not expressed in the normal genome (90). Personalized neoantigens have unique mutations and are significantly different from patient to patient (91). Nucleophosmin1 (NPM1) mutations are present in approximately 30% to 35% of AML cases and regarded as an optimal immunotherapy target (89). NPM1-specific CD8^+^ T cells in the context of the HLA-A*02:01-restricted NPM1 epitope CLAVEEVSL were generated from healthy donors by Van der Lee et al. (44). This clone effectively lysed the primary AML blasts. Subsequently, a codon-optimized TCR was generated from these clones, and adoptive transfer of NPM1/HLA-A*02:01-specific TCR-transduced T cells specifically killed both AML cell lines and primary AML blasts and controlled tumor outgrowth and prolonged survival in a xenograft model (44). These studies may suggest a role for shared neoantigens in TCR-based immunotherapy of AML and other hematologic malignancies.

Another neoantigen that is critical in leukemogenesis is a type-A variant of the fusion of core-binding factor β and myosin heavy chain 11 (CBFB-MYH11). The gene fusion event that leads to the formation of CBFB-MYH11 involves the inv(16) or t(16;16) cytogenetic abnormalities, and fusion occurs in approximately 90% of AML patients and 10% of individuals (92). Biernacki et al. generated CBFB-MYH11-specific CD8^+^ T cells in the context of CBFB-MYH11 and HLA-B*40:01-restricted T cells from healthy donors (45). A high-avidity CD8^+^ T cell clone showed the potential to kill relevant AML cell lines and primary human AML cells *in vitro* and *in vivo*. The construction of high-avidity TCR-specific CBFB-MYH11/HLA-B*40:01 T cells from this clone also demonstrated highly effective antileukemia activities *in vitro* and *in vivo*. The study concluded that the CBFB-MYH11 fusion neoantigen is immunologically targeting AML-initiating fusions. This study may represent the first critical step toward developing TCR-T cell immunotherapy targeting fusion gene-driven AML.

#### Other Antigen-Specific TCR-T Cells

Murine double minute 2 (MDM2) is an oncoprotein that is a potential inhibitor of wild-type p53 (wtp53) and can induce cell proliferation and promote cell survival (93). The MDM2 oncoprotein is overexpressed in several tumors, including hematopoietic malignancies (47, 94), and it has found to be an ideal target for AML immunotherapy. Thomas et al. generated MDM2-specific high-affinity TCR redirecting CTL in the context of HLA-A*02:01 for targeting leukemia. MDM2-specific TCR-CTL efficiently killed several human tumor and leukemia targets (47). Hyaluronan-mediated motility receptor (HMMR/Rhamm), a novel hyaluronan receptor complex component, was first purified from the supernatants of murine cells in 1992 (95). HMMR is broadly expressed in the neural crest and during embryogenesis, but its expression is limited to adult bone marrow (BM), thymus, and tonsils and in the placenta (46). It became an attractive target for cancer immunotherapy due to its overexpression in several tumors, including AML (46, 96). HMMR-specific TCR-T cells demonstrated high efficacy in killing AML *in vitro* and *in vivo*, and treating mice with HMMR-specific TCR-T combined with interleukin (IL)-15 exhibited potent efficiency in eliminating tumors and prolonged survival of AML-bearing mice (46). Human leukocyte antigen-DP β1 (HLA-DPB1) is a class of major histocompatibility complex (MHC)-II. The use of HLA-DPB1 in unrelated donor hematopoietic stem cell transplantation has been shown to improve outcomes in patients with leukemia relapse (97). Due to a common linkage imbalance between HLA-DR, HLA-DQ, and HLA-DP, approximately 80% of 10/10 matched unrelated donor-patient pairs are mismatched for one or both HLA-DPB1 alleles. Therefore, HLA-DPB1 mismatches predict a significantly lower risk of leukemia relapse (98). Herr et al. (98) generated AML-reactive CD4 CTL by stimulation of CD45RA-selected naive-enriched CD4 T cells of unrelated stem-cell donors with AML blasts of 10/10 HLA-matched patients. HLA-DPB1-mismatch-specific CD4 CTL effectively lysed HLA-DPB1 mismatch-expressing AML blasts and effectively eliminated human AML blasts in a xenograft model (98). Consistent with this study, Klobuch et al. have generated the HLA-DPB1-specific T-cell receptors from HLA-DPB1 mismatch-reactive allogeneic donor CD4 T-cell clones. They subsequently genetically optimized the receptor to enhance TCR expression and increase its activity against AML (49). HLA-DPB1-specific TCR-transduced CD4^+^ T and CD8^+^ T cells were strongly effective against primary AML blasts *in vitro*; however, *in vivo*, only DPB1 TCR-CD4^+^ T cells showed high-efficacy in the eradication of AML blasts in xenograft NOD SCID gamma mice (49).

### TCR-T Cell-Based Immunotherapy for AML in Clinical Studies

Adoptive transfer of antigen-specific TCR-T cells has demonstrated remarkable clinical outcomes in treating patients with relapsed or refractory AML; particular success has been seen with WT1-specific TCR-T cells (28, 29). The first human confirmation of the utility of WT1-specific TCR-transduced autologous T cells in the context of HLA-A*24:02 for treatment of refractory AML or high-risk MDS came in clinical trial UMIN000011519 (29). Among the eight patients enrolled in this study, two showed decreased blast counts in bone marrow, which predicted a regression from leukemia. Moreover, the WT1-specific TCR-T cells showed persistence in five patients, and four out of these five patients survived for more than 12 months. None of the patients experienced the adverse events related to toxicity in normal tissues (29).

HLA-A*0201-restricted WT1-specific donor-derived CD8^+^ cytotoxic T-cells (CTLs) for treating high-risk or relapses of 11 patients with leukemia, including those with AML, was reported early by Chapuis et al. in clinical trial NCT00052520 (99). Transduction of the cells led to demonstrated clinical responses in two patients: one patient experienced reduction of advanced progressive disease and another experienced prolonged remission. In addition, three patients at high risk for relapse post-HSCT survived without leukemia relapse or GVHD (99). Subsequently, Chapuis et al. continued to generate high-affinity WT1-specific TCR from HLA-A*02:01 healthy donor repertoires and cloned the TCR into Epstein–Barr virus (EBV)-specific donor CD8^+^ T cells to reduce GVHD and to enhance the transferring of T-cell survival (28). In clinical trial NCT01640301, 12 patients with relapsed or high-risk AML received allogeneic high-avidity WT1-specific TCR-T cells prophylactically. Interestingly, no toxicity was observed after the patients received adoptive transfer of WT1-specific TCR cells. The adoptive transfer of WT1-specific TCR-T cells led to 100% relapse-free survival at a median of 44 months, as compared with the control group with similar risk AML, which experienced approximately 54% RFS (28).

Moreover, a second study of WT1-specific TCR-transduced autologous T cells in the treatment of patients with high-risk AML and other myeloid malignancies has been reported by Morris et al. (30). In clinical trial NCT02550535, a total of 10 patients, including 6 AML, 3 MDS, and 1 tyrosine kinase inhibitor (TKI)-resistant CML, received the gene-modified T cells. No severe adverse events were associated with on-target, off-tumor toxicity in the ten patients treated with autologous WT1-specific TCR-T cells. Notably, seven out of ten patients who received the autologous WT1-specific TCR-T cells proliferated *in vivo* and persisted through the 12 month study period (30). Currently, a phase I/II clinical trial (NCT04284228), studying WT1, PRAME, and cyclin A1-specific stem cell donor CD8^+^ T cells in the context of HLA-A*02:01 (NEXI-001 T-cell product), is still enrolling. In addition, several studies of the WT1 antigen target and other antigen-specific autologous/allogeneic TCR-T cells also have been registered on ClinicalTrials.gov, including HA-1 (allogeneic, NCT03326921; autologous, NCT04464889) and PRAME (autologous, NCT03503968) (**Table 2**).

**Table 2:** Clinical studies of adoptive transfer of antigen-specific TCR-T cells against AML.

Identifier	TCR-T therapy	Leukemia	Phase	Outcome measures	Status	Locations
NCT02550535	Autologous WT1 TCR-T cells	■ Myelodysplastic syndromes; ■ Acute myeloid leukaemia	Phase 1	■ Safety following gene-modified WT1 TCR T-cell therapy as measured by suspected unexpected serious adverse reactions (SUSARS); ■ Proportion of subjects achieving 1 or more IWG response criteria following gene-modified WT1 TCR T-cell therapy; ■ Safety and tolerability of gene-modified WT1 TCR therapy as measured by clinical laboratory parameters and adverse events. ■ Among 10 patients (6 AML, 3 MDS, and 1 TKI-resistant CML) enrolled in the study, All 6 AML patients survived, at last, follow-up (median 12 months) and median 3 months in the 3 patients with MDS. 3 deaths: 2 from disease progression and 1 from other causes.	Completed	■ AZ St. Jan Brugge-Oostende AV Brugge, Belgium ■ UZ Leuven Leuven, Belgium ■ Uniklinikum Dresden, Germany
			Phase 2			
UMIN00001159	Autologous WT1 siTCR-T cells	■ Acute myeloid leukemia; ■ Myelodysplastic syndromes	Unknown	■ No adverse events of normal tissue were seen. ■ 2 patients showed transient decreases in blast counts in bone marrow, which was associated with recovery of hematopoiesis.	Completed	■ Mie University Hospital, Japan ■ Ehime University Hospital, Japan ■ Fujita Health University Hospital, Japan ■ Nagoya University Hospital, Japan
NCT01621724	Autologous WT1 TCR-T cells	■ Acute myeloid leukemia; ■ Chronic myeloid leukemia	Phase 1	■ Identify organ toxicities and other side effects ■ Transduction efficiency and TCR expression on TCR-transduced cells ■ WT1-specific immune responses of TCR-transduced T cells	Completed	■ University Hospitals Bristol NHS Foundation Trust Bristol, UK ■ University College London Hospitals NHS Trust London, UK, NW1 2PG
			Phase 2			
NCT01640301	Allogeneic WT1 TCR-T cells	■ Recurrent adult acute myeloid leukemia; ■ Recurrent childhood acute myeloid leukemia; ■ Secondary acute myeloid leukemia	Phase 1	■ Antileukemic potential efficacy, in terms of duration of response (Arm II). ■ Efficacy, in terms of relapse rate (Arm I). ■ Incidence of chronic graft versus host disease (GVHD) (Arm I).	Active, not recruiting	■ Fred Hutch University of Wash ington Cancer Consortium Seattle, Washington, USA
			Phase 2			
NCT04284228	Allogeneic WT1/PRAME/Cyclin A1-antigen-specific CD8^+^ T cells (NEXI-001 T-cell product)	■ Acute myeloid leukemia; ■ Myelodysplastic syndrome	Phase 1	■ Adverse events of special interest (AESIs) events of dose-limiting toxicities (DLTs) ■ AESI events of infusion-related reactions and cytokine release syndrome (CRS) ■ Survival, including median progressive-free survival (PFS), overall response rate (ORR), overall survival (OS).	Recruiting	■ City of Hope Comprehensive Cancer Center Duarte, California, USA ■ Advent Health Medical Group Blood & Marrow Transplant Orlando, Florida, USA ■ Karmanos Cancer Institute Detroit, Michigan, United States
			Phase 2			
NCT03503968	Autologous PRAME TCR-T cells (MDG1011 cell product)	■ High-risk myeloid; ■ Lymphoid neoplasms (including relapse AML after allo-HSCT)	Phase 1	■ Adverse events and dose limiting toxicities (safety and tolerability). ■ Maximum tolerated dose (MTD) and/or recommended phase II dose (RP2D) of MDG101. ■ For feasibility: percent of all subjects who receive the planned target dose of MDG1011.	Recruiting	■ University Hospital Dresden, Dresden, Germany ■ University Hospital Erlangen, Erlangen, Germany ■ University Hospital Frankfurt, Frankfurt, Germany
			Phase 2			
NCT03326921	Allogeneic HA-1 TCR-T cells	■ Juvenile myelomonocytic leukemia ■ Recurrent acute biphenotypic leukemia ■ Recurrent acute undifferentiated leukemia	Phase 1	■ Feasibility of manufacturing minor H antigen (HA-1) T-cell receptor (TCR) CD8+ and CD4+ T cells. ■ Feasibility of administering minor H antigen (HA-1) T-cell receptor (TCR) CD8+ and CD4+ T cells. ■ Incidence of dose-limiting toxicities of HA-1 T-cell receptor (TCR) T cells.	Recruiting	■ Fred Hutch University of Washington Cancer Consortium Seattle, Washington, United States
NCT04464889	Autologous HA-1 H TCR-T cells	■ Acute myeloid leukemia ■ Myelodysplastic syndromes	Phase 1	■ Safety and tolerability of HA-1H TCR-transduced T cells: incidence and severity of adverse events. ■ Maximum tolerated dose (MTD) of HA-1H TCR-transduced T cells. ■ Recommended phase 2 doses (RP2D) of HA-1H TCR-transduced T cells.	Active, not recruiting	■ Leiden University Medical Centre Leiden, Zuid Holland, Netherlands

## The Challenges of Adoptive TCR-T Cell Immunotherapy for AML

Several TCR-T cell immunotherapies for AML are in use in the clinic, but some obstacles relevant to this approach need to be overcome to enhance the clinical benefits. The benefits of TCR-T cell therapy for AML may remain limited unless a thorough evaluation is made of its on-target/off-tumor toxicity, its dose-related toxicity, the persistence of TCR-T cells *in vivo*, and the chance of immune escape by AML after TCR-T administration.

### On-Target, Off-Tumor Toxicity

One concern of therapies involving the adoptive transfer of antigen-specific TCR-T cells is on-target/off-tumor toxicity that may occur if nontarget tissues, such as those of the hematopoietic system, are recognized as targets. This possibility is exacerbated when antigen targets are expressed in normal tissues. Two clinical trials have reported the occurrence of off-target toxicity-related adverse effects upon adoptive transfer of autologous TCR-T cells, including neurotoxicity and cardiac toxicity (100, 101). Two patients treated with high-affinity TCR-T cells, for example, showed symptoms of cardiogenic shock and died within a few days of T-cell infusion. Here, the TCR-T cells recognized a similar peptide epitope derived from the entirely unrelated protein titin expressed in cardiac tissue (100). Similarly, two out of nine melanoma patients treated with TCR-T cells that recognized epitope MAGE-A3/9/12 lapsed into comas. They died after T-cell infusion due to the expression of MAGE-A12 in the human brain, which may have been attacked by the MAGE-specific TCR-T cells (101).

Adverse events of on-target toxicity have also been reported in metastatic melanoma treated with high-avidity TCR-transduced autologous T cells specific for MART-1 and gp100 in the context of HLA-A*0201. After infusion, these patients showed a therapeutic response but experienced adverse events, including skin rash, hearing loss, and uveitis (102). Severe inflammatory colitis has been demonstrated in colon cancer patients who received adoptive transfer of carcinoembryonic antigen-specific autologous TCR-T cells in the context of HLA-A*0201 (103).

Several clinical trials reported high efficacy and safe use of NY-ESO-1 antigen-specific TCR-T in clinics (25–27, 104). AML blasts have a low level of NY-ESO-1 expression due to the silencing of CTA expression *via* promoter methylation (105, 106). Several groups have reported that treatment of AML with the hypomethylating agent decitabine *in vitro* and *in vivo* resulted in upregulation of the expression of CTAs such as NY-ESO-1 (104, 106, 107). NY-ESO-1 vaccination combined with decitabine in the targeting of AML has shown impressive results in clinical studies (104). These clinical results evoked the idea that demethylating agents could promote NY-ESO-1-specific TCR-T cells to target and kill AML. Accordingly, our group recently demonstrated that the use of NY-ESO-1_157–165_ HLA-A*02:01-specific TCR-T cells against decitabine-induced AML efficiently lysed AML cell lines and primary AML blasts and targeted AML in a xenograft model (data not shown). Therefore, NY-ESO-1-specific TCR-T combined with decitabine could be a potent approach for future clinical investigations in patients with relapsed or high-risk AML.

T cells referred to as γδ T cells, which represent from 1% to 10% of peripheral blood T cells, express a γδTCR that is not able to form a complex with αβ TCRs (108). Therefore, strategies designed to redirect γδ T cells with αβ TCR or to redirect αβ T cells with γδTCR may overcome the limitation of TCR mispairing, which can risk mediating self-reactivity (42, 109). Accordingly, αβ TCR-specific mHag HA-2-transduced γδ T cells have shown high-potency and antigen-specific killing or primary leukemia blasts with a good safety profile (42). The γδ T cells transduced with αβ TCR and CD8 receptor in the context of HLA-A*02:01-restricted HA-2 showed high levels of antigen-specific cytolytic activity against HA-2-expressing AML and CML blasts (42). In addition, transduction of γδ T cells with αβ TCR and CD4 receptor in the context of HLA class II-restricted human Y chromosome antigen DBY-TCR also showed high cytotoxicity against target cells (42). Some clinical trials of αβ TCR-modified allogeneic γδ T cells have been described in a literature review (110).

Alternatively, it is possible to redirect the αβ T cells (T cells) with γδ TCR cells (109). Redirecting CD4^+^ and CD8^+^ αβ T cells with γ9δ2TCR also has been shown to lead to efficient killing of primary AML *in vitro* and in a xenograft model (109). Vyborova et al. have successfully generated γ9δ2TCR clones from healthy donors, and the clones mediated antitumor responses against malignant cancers. In addition, the γ9δ2TCR-transduced αβ T cells, a product known as TEG001, were shown to recognize the butyrophilin subfamily 2 member A1 peptide antigen, and demonstrated functional enhancement activity against leukemia *in vitro* and *in vivo* (111, 112). Analysis of TEG001 is underway in a first-in-human clinical trial (NTR6541).

### Dose-Related Toxicity

Dose-related toxicity has been reported in some patients receiving a high concentration of MAGE-A3-specific TCR-transduced autologous T cells (101). Patients developed neurologic toxicity after receiving a total dose higher than 6.73 × 10^10^ cells (101). In a phase I clinical trial (NCT02858310), one patient with metastatic human papillomavirus (HPV)-associated epithelial cancer experienced dose-limiting toxicities (DLTs) at dose level 3 after receiving 1 × 10^11^ HPV E7-specific autologous TCR-T cells (E7 TCR-T) (113). Adverse events and DLTs were also identified in patients treated with a higher dose of autologous genetically modified MAGE-A10^c796^TCR-T cells (114).

Several clinical studies have also revealed issues with dose-related toxicity of CD19 CAR-T cells (115–117). The dose-related toxicity may be associated with cytokine release syndrome triggered by the administration of higher doses of CAR-T cells or the achieving of higher cell numbers due to *in vivo* proliferation of CAR-T cells (118). Toxicity induced by the administration a large number of cells may occur immediately after transfusion and may be caused by the triggering of cytokine release by the recognition of low levels of antigen on the surfaces of cells. In one related report, toxicity manifest as severe encephalopathy of was observed in 3 out of 28 patients who received doses between 1.0 and 5.0 × 10^8^ cells in an anti-CD19 CAR-transduced autologous T-cell (CTL019) treatment. One out of the three patients who experienced this encephalopathy died due to follicular lymphoma progressive neurologic deterioration (119). In a phase I clinical trial (NCT01593696), a dose-escalation experiment was conducted to study treatment of children and young adult patients with ALL and non-Hodgkin’s lymphoma (NHL) with autologous transfusion of doses of 1.0 × 10^6^/kg (dose 1) or 3.0 × 10^6^/kg (dose 2) CD19 CAR-T cells (117). Two of twenty-one patients who received dose 2 demonstrated dose-limiting toxicity, specifically manifested as grade 3 and grade 4 cytokine release syndrome. Other high-grade toxicities resulting from various doses of CD19 CAR-T cells have been summarized elsewhere (116).

Therefore, dose optimization of TCR-T cells is necessary to overcome the limitation of adverse events related to dose toxicity in clinical applications. Accordingly, in clinical trial NCT02858310, Nagarsheth et al. (113) have demonstrated dose optimization of E7 TCR-transduced autologous T cells to treat HPV-related cancers. The patients were treated with various doses (1 × 10^9^, 1 × 10^10^, and 1 × 10^11^) of TCR-T cells. This study suggested that administering the maximum amount of 1 × 10^11^ TCR-T cells was not limited by toxicity in most patients. Other clinical studies, including NCT03503968 and NCT04464889, are ongoing to evaluate the dose titration of autologous TCR-T cells to target myeloid leukemia and other hematopoietic malignancies for avoiding adverse events or dose-related toxicity.

### Persistence of TCR-T Cells *In Vivo*

The localization and persistence of adoptively transferred therapeutic T cells are critical factors in cancer elimination and relapse prevention (29, 120). However, a challenge associated with ACT is the short lifespan of T cells, which limits the long-term persistence and expansion of these cells *in vivo*, therefore reducing the therapeutic efficacy. The enhanced persistence of T cells *in vivo* can be achieved through several strategies, including genetic modification of T-cell signaling and stimulation of T cells with cytokines or drugs. For example, the proliferation and persistence of TCR-T cells can be boosted by inserting the intracellular domain (ICD) of moieties that activate T-cell signaling (CD28 or 4-1BB) into CD3ζ instead of modifying TCR affinity. These modified TCR-T cells have been demonstrated to have increased efficacy with enhanced proliferation and long-term lifespans *in vivo* (121, 122).

Administration of cytokines together with antigen-specific T cells has been shown to enhance T-cell persistence and to lead to the production of T memory stem (TSCM) cells (99, 123–126). Exposure of WT1 antigen-specific donor-derived CD8^+^ T cells to IL-21 resulted in prolonged remission of patients with leukemia. In all these patients with leukemia, the T cells remained present and were maintained, and their long-term *in vivo* phenotypic and functional characteristics evolved with long-lived memory T cells (99). Recently, an animal model has been used to show that treatment of CAR-T cells with low-dose decitabine led to high efficacy and persistent antitumor activity (127). Thus, TCR-T cells treated with low-dose decitabine may also increase phenotypic markers of T memory stem cells.

### Mechanisms of Immune Evasion in AML

**Figure 2:**
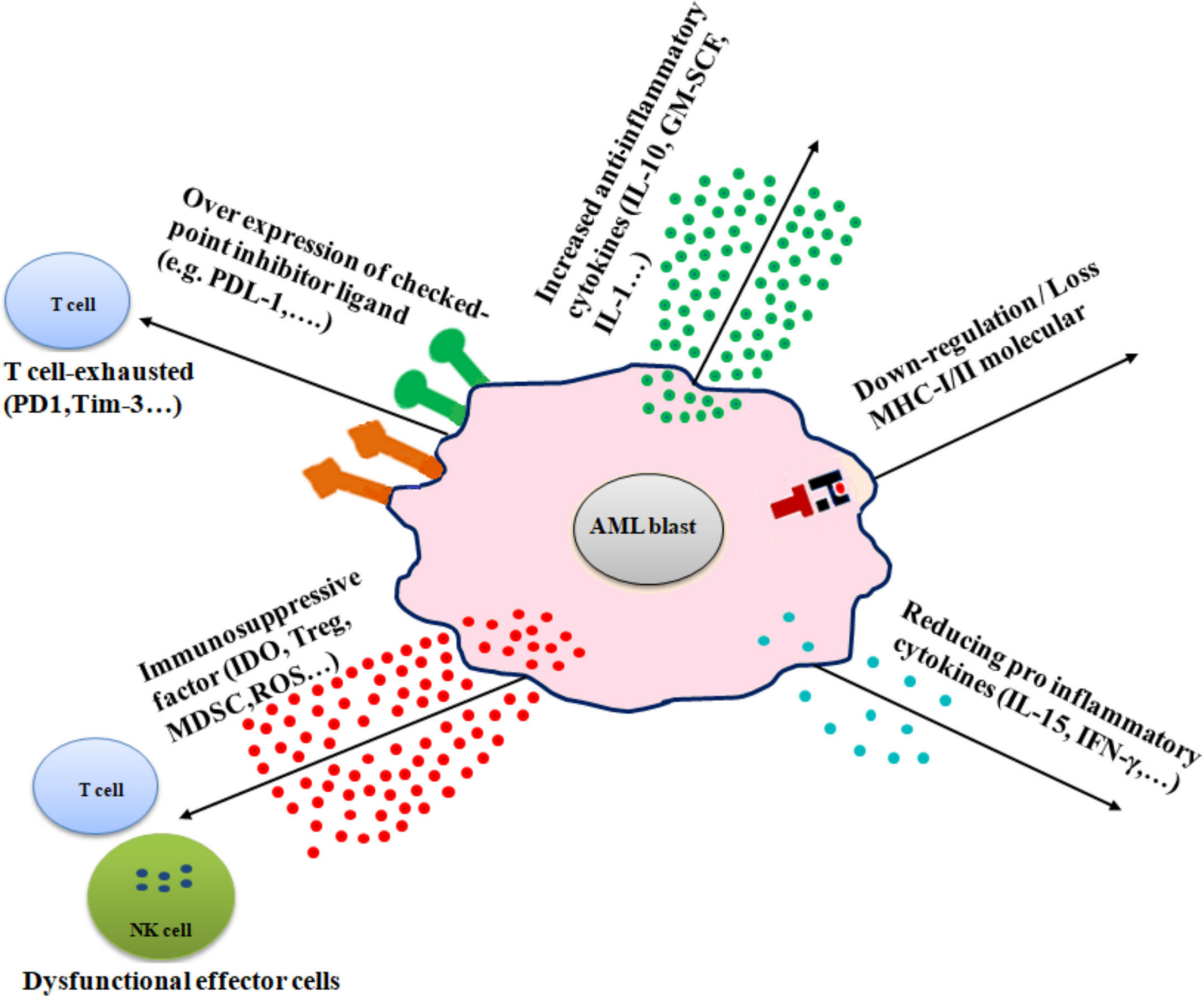
Diverse immune escape mechanisms of AML from immune effector cells. AML cells use several mechanisms to prevent immune effector cell patrolling, including downregulation or loss of MHC molecules (MHC-I/MHC-II), increased inflammatory cytokines (e.g., IL-10, IL-1, and GM-SCF), overexpression of checkpoint inhibitor ligand (e.g., PD-L1 and B7-H3), release of immune-suppressive factors (e.g., IDO, Treg, and MDSC), and reduction of proinflammatory cytokines (e.g., IL-15 and IFN-γ).

Several mechanisms are involved in immune evasion in AML, including (1) alteration of antigen expression by downregulation or loss of MHC molecules, (2) overexpression of immune checkpoint inhibitors, (3) production of immunosuppressive factors, (4) excessive secretion of anti-inflammatory cytokines, and (5) and reducing proinflammatory cytokines **(** **Figure 2** **).**

#### Alteration of Antigen Expression

The elimination of AML blasts by allo-HSCT depends on the recognition of peptides presented by MHC molecules on the cell surface. AML relapse due to the loss of the mismatched HLA haplotype has been observed in the HSCT of donor T cells or bone marrow transplantation (128, 129). A case study reported a patient with leukemia who had two occurrences of leukemia relapse due to loss of mismatched HLA after receiving allo-HSCT (130). In the context of TCR-T therapy, adoptive transfer of NY-ESO-1 antigen-specific TCR restricted to HLA-A*02:01 against multiple myeloma has shown recurrence after the treatment. In this case, the analysis of myeloma cells demonstrated that tumor relapse was associated with definite loss of HLA-A*02:01 expression from the cell surface (131). Moreover, several studies have shown that AML relapse was associated with downregulation of MHC class II after allo-HSCT or posttransplantation (132–134). Because members of the interferon family (IFN), such as IFN-α, IFN-β, and IFN-γ, play an important role in the promotion of MHC-I expression (135), a strategy based on insertion of IFN-γ into the C-domain of a TCR may overcome the limitation of MHC molecule down-regulation. Tumor targeting of antibody-IFN-γ fusion proteins has shown highly potent anticancer activities associated with a receptor-trapping mechanism (136).

#### Overexpression of Immune Checkpoint-Related Proteins

By upregulating ligands that activate immune checkpoints, AML cells can induce exhaustion in T cells and can thus escape from immune surveillance mechanisms (137). It has been shown that an increased level of expression of programmed cell death protein 1 (PD-1) in CD8^+^ and CD4^+^ T cells after allo-HSCT results in T-cell exhaustion, leading to AML relapse (138, 139). In the setting of relapse post-allo-HSCT setting, a study of patient samples showed the upregulation of several ligands on AML blasts, including PD-1 ligand (PD-L1), B7-H3, and poliovirus receptor-related 2 (PVRL2) (133, 140). Overexpression of PD-1 has been reported in patients with metastatic melanoma who received adoptive transfer of MART-1 antigen-specific T cells (141). Exhaustion of tumor-specific CD8^+^ T cells has been investigated in metastases with melanoma patients caused by upregulation of several inhibitory receptors, including PD-1, CTLA-4, and Tim-3 (142). Therefore, strategies based on blocking inhibitory receptors could represent practical therapeutic approaches by stimulating the synergy of the antileukemia immune response. Treatment of AML relapse with antibodies blocking inhibitory receptors has exhibited remarkable results with high effectiveness in the clinic, as described and reviewed elsewhere (137, 143).

#### Immunosuppressive Factors

Multiple immunosuppressive factors, such as reactive oxygen species (ROS), indoleamine 2,3-dioxygenase (IDO), regulatory T cells (Treg), and myeloid-derived suppressor cells (MDSC), have been found to be involved in immune escape in AML. It has been found that immature myeloid cells derived from tumor-bearing mice increased ROS levels, inhibiting the cytotoxicity activity of CD8^+^ T cells as compared with tumor-free animals (144). Moreover, a study of human peripheral blood and bone marrow from AML patients demonstrated that monocytic AML cells secreted ROS to kill T cells and natural killer cells by activating poly-[ADP-ribose] polymerase-1-dependent apoptosis (145). In addition, the expression of enzymes involved in the production of immunosuppressive products such as IDO is increased in AML patients and can hamper T-cell responses through the induction of high expression of Treg (146).

Low-risk MDS is related to the proliferation of autoimmunity-associated T helper 17 (Th17) cells, whereas a decreased number of Th17 cells and the expansion of Treg are regarded as indicators of high-risk MDS (147). In one study, a positive correlation of the number of Tregs and MDSCs was observed in patients with high-risk MDS but not in those with low-risk MDS, suggesting a role of MDSCs in the *in vivo* expansion of Tregs in MDS and subsequent disease progress (148). The development of the disease can be explained by increased levels of intracellular cytokines IL-10 and TGF-β in MDSCs (148). A higher level of MDSCs in bone marrow may be regarded as a prognostic factor for AML (149, 150). Alternatively, recent studies indicated that MDSC-like blasts from bone marrow mononuclear cells of AML patients could increase the levels of arginase-1 (ARG1) and inducible nitric oxide synthase (iNOS) that restrain CD8^+^ T-cell proliferation and induce T-cell apoptosis (151). Decreased MDSCs have enormously enhanced the TK/Flt3L gene-induced tumor-specific CD8 T-cell response to patients with gliomas (152). Nagaraj et al. have demonstrated that coculture of antigen-specific CD8^+^ T cells with peptide-loaded MDSCs disrupted signaling downstream of TCR (153).

#### Excessive Secretion of Anti-Inflammatory Cytokines

Increased levels of anti-inflammatory cytokines have been identified in the plasma of AML patients (154–156). It is acknowledged that leukemic cells can freely escape from immune surveillance by producing anti-inflammatory cytokines such as TGF-β (157, 158). These studies have reported a dual biological effect of IL-10, including tumor-promoting and antitumor functions, with respect to cancer (159, 160). As a tumor progresses, high levels of IL-10 exhibit powerful immunosuppressive effects through inhibiting the proliferation of T cells and the production of cytokines such as IFN-γ and IL-2 (159). IL-10 suppression was found to enhance the antitumor activity against CLL (161). In addition, combining T-cell therapy with treatments targeting immune cell PD-1 showed high efficacy against leukemia *via* the production of more IFN-γ, the increasing of cytolytic functions, and the increasing of memory CD8^+^ T cells (161).

#### Reducing Proinflammatory Cytokines

Proinflammatory cytokines, such as IL-15 and IFN-γ, that are produced by myeloid or lymphoid progenitor cells play an essential role in eliminating leukemia cells (13, 162). Low serum levels of IL-15 in patients with leukemia early post-allo-HSCT were associated with relapse of the disease (163). Moreover, combining NK cells and exogenous IL-15 was demonstrated to enhance the immune effector cells to eradicate leukemia in post-allo-HSCT in a mouse model (164). In a phase I clinical trial (NCT01885897), administration of an IL-15 superagonist complex (ALT-803) significantly improved CD8^+^ T cell and NK cell functions in relapsed patients with leukemia post-allo-HSCT (165). Thus, high levels of IL-15 in the microenvironment may contribute to suppressing leukemia, since it can boost the effector cells. The strategy to modify TCR-T cells with proinflammatory cytokines such as IL-18 or IL-12 has been found to increase persistence and high antitumor efficacy with a good safety profile in animal models (166, 167).

Low levels of IFN-γ cytokine secretion also have been observed in patients with leukemia. Analyses of clinical samples from B-lineage ALL patients showed that high-risk groups were associated with low IFN-γ expression, which causes leukemia to evade immune cells (168). In addition, leukemia cells may bypass the immune system by suppressing inflammatory growth factors, including IL-1b and G-CSF (13).

## Comparison Between TCR-T and CAR-T Cell Therapies for AML

Although CAR-T and TCR-T cells have been successfully used as a paradigm-shifting in cancer immunotherapy for treating several cancers, each approach has advantages and disadvantages (**Table 3**). The significant benefit for TCR-T over CAR-T is the ability to target peptide proteins intracellularly or cell surface proteins (169). CAR-T can only recognize target peptides on the cell surface antigens. Most proteins have been reported to express intracellular cells instead of the small number of proteins (~28%) expressed on the cell surface, making them unable to be selected as antigen for CARs (170). TCRs also have structural advantages than CARs, including more subunit receptors (ten subunits vs. one subunit), more costimulate receptors, and less dependent on antigen requirement for T-cell activation (one vs. 100) (171).

**Table 3:** Comparison between CAR-T and TCR-T cell therapies.

	Advantages	Disadvantages
TCR-T	■ Recognizing antigens expressed on the cell surface or intracellular antigens	■ Recognizing antigen targets in MHC-restricted manner
	■ High sensitivity and more specificity	■ TCR-T is still underway the phase of clinical trials
	■ Structural advantages: more subunit receptor, more costimulate receptor, and less dependence on antigens	■ Possible toxicity due to misparing between exogenous with endogenous TCR or on-target/off-tumor toxicity dose-related toxicity
	■ Several AML specific antigens have been reported (e.g., WT1 and neoantigens)	
CAR-T	■ Enables antigen targets without MHC restriction.	■ Targeting antigens expressed on the cell surface
	■ FDA have been approved CAR-T therapy for several forms of cancers	■ Toxicity due to cytokine release syndrome
		■ Lack of AML-specific antigens. Common specific antigen found (e.g., CD19, CD33, and CD34)
		■ Less sensitivity and low specificity

AML has been reported to have lower mutational burden compared with solid cancers. Therefore, AML seems to possess relatively fewer neoantigens that can be targeted by CAR-T therapy compared with other malignancies (172). Unlike CAR-T, TCR-T has been shown expressing several mutational neoantigens in AML, as described in the following section. CARs have a higher affinity than TCRs but have less sensitivity than TCR in comparing the affinity of a single-chain TCR (Vβ-linker-Vα) with scFv that serves as a CAR-like receptor (use the same recognition domain) (173). Thus, TCRs offer an expanded capacity to address a larger variety of carcinomas.

One major obstacle is that TCR-T cell therapy is restricted to MHC proteins of certain HLA alleles. Thereby, each TCR-T cell treatment is only suitable to patients who have a matched HLA genotype. This characteristic decreased the number of eligible patients enrolled in the TCR-T clinical trials. In contrast, CAR-T cells are MHC independent and can be applied in patients of all HLA types (169).

Both CAR-T cells and TCR-T cells have on-target, off-tumor (i.e., antigen on normal tissue) toxicities resulting from the target antigens expressed on nonmalignant cells. B-cell aplasia (43), cytokine release syndrome (87, 88), and central nervous system toxicity (88, 89) have been observed in patients receiving CAR-T cells. Although TCR-T cells are designed to redirect antigen reactivity and maintain specificity, preclinical and clinical data have demonstrated the potential for TCR-T cells to exhibit on-target, off-tumor recognition or off-target cross-reactivity (i.e., related or unrelated antigen on target or nontarget tissue). In an early clinical trial, two treated patients developed cardiogenic shock and died within a few days of anti-MAGE-A3 affinity-enhanced TCR-T cells, due to off-target reaction directed against an unrelated protein (titin) in striated muscles (100). Thus, more advanced methods to predict or experimentally probe the risk of off-target toxicities are needed for ACT therapies prior to clinical trial.

## Conclusions and Future Prospects

Adoptive transfer of antigen-specific TCR-T cells is a promising tool for AML immunotherapy due to the ability of these cells to distinguish between normal and malignant tissues. Several clinical studies have demonstrated significant clinical responses with safe profiles and have improved survival, particularly WT1-specific TCR-T cell therapy. Moreover, the analyses of multiple antigen-specific TCR-T cells also are underway in a clinical trial for AML immunotherapy (**Table 2**). However, there are several limitations to the adoptive transfer of antigen-specific TCR-T cells in AML therapy. Although clinical trials of TCR-engineered T cells demonstrated impressive results and efficacy, this treatment strategy is disrupted by treatment-related on-target/off-tumor toxicity or dose-related toxicity. Many potential antigen targets of CTAs, including NY-ESO-1, PRAME, and MAGE, are rarely expressed in AML. Moreover, the persistence of ***in vivo*** TCR-T cells remains a hurdle in AML immunotherapy due to inhibition of T-cell expansion by AML blasts. As a heterogeneous and complex disease, AML evaded the immune cells by several immunosuppressive mechanisms.

Some challenges need to be overcome to ensure the safe and effective use of TCR-T cells in AML therapy. To overcome the limitations of on-target/off-tumor toxicity, choosing an appropriate antigen target is an effective strategy for eliminating malignant cancers. In this respect, tumor-restricted CTAs may be considered potentially safe target antigens. As mentioned above, combining treatment with DNA hypomethylation agents also can induce the expression of several tumor antigens to engage cognate TCRs, thereby potentially activating the adoptively transferred T cells. Dose optimization of TCR-T cells also can prevent patients from experiencing dose-related toxicities. In addition, other improvements have also expanded the persistence of TCR-T cells *in vivo*, including combining the treatment with exogenous cytokines (e.g., IL-21, IL-7, and IL-15) during cell expansion and with demethylating agents such as decitabine. Alternatively, the addition of genetically engineered constitutively signaling cytokine receptors in TCR-T cells also can lead to secretion of immunostimulatory cytokines such as IL-15 and IL-12. The use of adoptive transfer of TCR-T cells in combination with immune checkpoint blockade also can provide a novel strategy to improve immunotherapies. Thus, adoptive transfer of TCR-T therapy is a promising treatment technique for AML immunotherapy, but further investigations are warranted.

## Author Contributions

SK and XG contributed to the conception and design of the article. SK was accountable for the drafting and writing of the manuscript. XG, YL, JQ, ZH, and XM made critical revisions of the manuscript for important intellectual content. XG and LY were the supervisors. All authors listed have made a substantial, direct, and intellectual contribution to the work and approved it for publication.

## Funding

This work was supported by grants from the Chinese National Major Project for New Drug Innovation (2019ZX09201002003), National Natural Science Foundation of China (82030076, 82070161, 81970151, 81670162, 81870134 and 81900474), Shenzhen Science and Technology Foundation (JCYJ20190808163601776, JCYJ20200109113810154), Shenzhen Key Laboratory Foundation (ZDSYS20200811143757022), Sanming Project of Medicine in Shenzhen (SZSM202111004), and Natural Science Foundation of Shenzhen University General Hospital (SUGH2019QD012).

## Conflict of Interest

The authors declare that the research was conducted in the absence of any commercial or financial relationships that could be construed as a potential conflict of interest.

## Publisher’s Note

All claims expressed in this article are solely those of the authors and do not necessarily represent those of their affiliated organizations, or those of the publisher, the editors and the reviewers. Any product that may be evaluated in this article, or claim that may be made by its manufacturer, is not guaranteed or endorsed by the publisher.

## Abbreviations

allo-HSCT, allogeneic hematopoietic stem cell transplant; ATL, adult T-cell leukemia/lymphoma; CAR-T, chimeric antigen receptor modified-T cells; CLL, acute lymphoid leukemia; CR, complete response; CTA, cancer-testis antigen; GM-SCF, granulocyte-macrophage colony-stimulating factor; HA-1, minor histocompatibility antigen; IDO, indoleamine 2,3-dioxygenase; IFN-γ, interferon gamma; IL-10, interleukin-10; IL-15, interleukin-15; IL-1, interleukin-1; LCLs, lymphoblastoid cell lines cells; MDS, myelodysplastic syndrome; MDSC, myeloid-derived suppressor cells; mHag, minor histocompatibility antigen; RFS, relapse-free survival; PBLs, peripheral blood lymphocytes; PD-L1, programmed cell death-ligand 1; ROS, reactive oxygen species; TCB, TCR-like T-cell bispecific antibody; TCR-T, T-cell receptor modified-T cells; TERT, telomerase reverse transcriptase; TKI, tyrosine kinase inhibitor; Treg, regulatory T cells.
